# My remembrances of H.G. Khorana: exploring the mechanism of bacteriorhodopsin with site-directed mutagenesis and FTIR difference spectroscopy

**DOI:** 10.1007/s12551-023-01046-9

**Published:** 2023-02-06

**Authors:** Kenneth J. Rothschild

**Affiliations:** Department of Physics, Department of Physiology and Biophysics, and Photonics Center, Boston University, 590 Commonwealth Avenue, Boston, MA 02215 USA

**Keywords:** Gobind Khorana, Bacteriorhodopsin (BR), Rhodopsin, Raman spectroscopy, FTIR difference spectroscopy, Integral membrane proteins

## Abstract

H.G. Khorana’s seminal contributions to molecular biology are well-known. He also had a lesser known but still major influence on current application of advanced vibrational spectroscopic techniques such as FTIR difference spectroscopy to explore the mechanism of bacteriorhodopsin and other integral membrane proteins. In this review, I provide a personal perspective of my collaborative research and interactions with Gobind, from 1982 to 1995 when our groups published over 25 papers together which resulted in an early picture of key features of the bacteriorhodopsin proton pump mechanism. Much of this early work served as a blueprint for subsequent advances based on combining protein bioengineering and vibrational spectroscopic techniques to study integral membrane proteins.

## Early encounters and motivations

I have extremely fond memories of Gobind, who greatly influenced my scientific career and life in general. He was not only an inspiration and mentor but a model for success in science. I got to know Gobind well in the 1980s and 1990s when our groups explored the mechanism of bacteriorhodopsin (BR) together. This involved using a combination of advanced genetic engineering and vibrational spectroscopy. Some of this work helped contribute to our current understanding of microbial rhodopsins. To a large extent this combination of molecular biology and vibrational spectroscopy was an early forerunner of future studies that continue to have lasting impact on understanding the molecular mechanism of proteins in general. Our interaction also led to close scientific collaborations and lasting friendships with many of the people that worked with Gobind as members of his group or collaborators. I also was greatly influenced by my interactions with Tom RajBhandary, an MIT Professor and close collaborator of Gobind’s throughout his career.

My first brief encounters with Gobind were during my graduate studies at MIT from 1969 to 1974 and afterwards as a post-doctoral Fellow in the Harvard-MIT Program in Health Sciences and Technology (HST) from 1975 to 1976 (Rothschild 2016). I worked closely during this period with my Ph.D. supervisor, Gene Stanley, a physicist and pioneer in statistical physics, on developing statistical/mechanistic models of selective ion transport in membranes and the basis for non-equilibrium linearity in membrane proteins (Rothschild and Stanley 1975, Rothschild et al. 1980a). Later in my graduate career I became interested in applying vibrational spectroscopic methods like Raman and FTIR spectroscopy to study model of membrane proteins, such as valinomycin and gramicidin (Rothschild et al. 1973; Rothschild and Stanley 1974) and later rhodopsins, the retinal based integral membrane proteins that serve as the light receptor in animal vision and microbial phototaxis as well as other functions such as light-driven proton transport (Rothschild et al. 1976; Rothschild and Clark 1979a, b).

As part of my education, I spent much of my time in the MIT Hayden library overlooking the Charles River and remember the day I discovered a book on molecular biology that described the pioneering work of Gobind on elucidation of the genetic code, a key milestone in the molecular biology revolution. This was a seminal moment for me and triggered my life-long interest in molecular biology. I subsequently attended a course on molecular biology with Harvey Lodish at MIT and James Watson at Harvard. I also worked for a year in Alex Rich’s lab, who had just solved the 3D crystallographic structure of a tRNA^Phe^. When Gobind arrived at MIT in 1970 from the University of Wisconsin, I began attended his lectures which were special events and followed closely his work on producing the first synthetic gene coding for alanine-tRNA, a hallmark of molecular cloning, synthetic biology and the dawn of the biotechnology industry.

In the 1970s Gobind turned his focus to understanding the molecular mechanism of membrane proteins after a sabbatical in Efraim Racker’s lab at Cornell. He wisely perceived that bacteriorhodopsin (BR), a membrane protein found in the purple membrane of *Halobacterium Salinarum*, was the ideal integral membrane protein to study and was ripe for a new approach using genetic engineering. This protein had initially been discovered by W. Stoeckenius’s group at UCSF and shown to possess many unique properties (Stoeckenius 1976). These include a 2D crystalline lattice, the ability to convert light energy into the movement of a proton across a biomembrane electrochemical gradient which powered the synthesis of ATP, a key tenant of the Mitchell Chemiosmotic hypothesis (Mitchell 1961), as well as possessing the same retinylidene chromophore as the visual pigment rhodopsin.

This matched closely my own growing interest in BR and animal rhodopsins which began in 1976 when we showed using Raman spectroscopy in collaboration with Wim DeGrip (working then as a post-doc in George Wald’s lab at Harvard) that the apoprotein of bovine rhodopsin (opsin) had primarily an α-helical structure (as opposed to early models which envisioned a gramicidin-like β-helical structure) (Rothschild et al. 1976). Richard Henderson and Nigel Unwin at the MRC had also shown the year before using electron diffraction of monolayers of the purple membrane that its core structure consisted primarily of a cluster of 7-tube like segments, presumably α-helical, oriented roughly perpendicular to the purple membrane plane (Henderson and Unwin 1975).

After I moved to Boston University in 1977 to establish a molecular biophysics lab focused on applying vibration spectroscopic techniques to membrane proteins, we were able to show using polarized FTIR spectroscopy in collaboration with Noel Clark, then at Harvard University, that the structure of both BR and rhodopsin consisted primarily of α-helices which were oriented perpendicular to the membrane plane in agreement with earlier high-resolution electron diffraction images map of BR (Rothschild and Clark 1979a, b; Rothschild, Sanches et al. 1980a, b).

In 1979, one of the first fruits of Gobind’s new focus on integral membrane proteins emerged when his group elucidated the amino acid sequence of BR using novel amino acid sequencing methods (Khorana et al. 1979). This was an important milestone since it represented one of the first known amino acid sequence of an integral membrane protein. Importantly, it revealed that embedded in the sequence were 7 hydrophobic segments which corresponded to the 7 transverse α-helices detected by electron diffraction. This helped establish the now well-known transverse α-helical motif found in all G-protein coupled receptors (GPCRs) as well as type I microbial rhodopsin such as BR and type II rhodopsins (visual pigments).

## Localizing the attachment site of retinal in BR

In the early 1980s an important question concerned the linkage site of the light absorbing retinal chromophore in BR. While it was known that the linkage involved the ε-amino group of a lysine residue (Lewis 1978; Argade et al. 1981), chemical evidence suggested two possible attachment sites, Lys41 and Lys216 (Katre et al. 1981). This result raised the possibility that during the BR photocycle the attachment site changed, thereby facilitating proton transport. My first collaboration with Gobind involved addressing this question (Rothschild et al. 1982). Gobind’s group in a groundbreaking discovery had shown that the BR could be cleaved with chymotrypsin yielding two intact fragments (C-1) (amino acids 72–248) and C-2 (amino acids 1–71) and the two fragments can then under certain conditions self-assemble into a reconstituted lipid bilayer membrane in a functional form (Huang et al. 1981). We took advantage of this property, which also involved a collaboration with Judy Herzfeld then at the Harvard Medical School, by reassembling the C1 and C2 fragments which had been derived from BR expressed in *H. Salinarum* in a stringent medium so one of the two fragments or both had N^15^ isotopically labeled lysines at the ε-amino group position. Since the C = N stretch mode of the Schiff base formed by the retinylidene chromophore and a lysine group underwent a downshift in frequency only when it was attached to the labeled fragment for both the BR_570_ and M_412_ states, we were able to show that the attachment site was not Lys-41 residing on the C-2 fragment for these states but C-1 which contained the second putative binding site, Lys-216. This conclusion was strongly reinforced by additional studies by Gobind’s group which showed that selective methylation of C-2 did not block reassembly with C-1, retinal regeneration or functional proton transport, whereas methylation of C-1 had the opposite effect (Huang et al. 1982).

## Combining site-directed mutagenesis and FTIR difference spectroscopy

One of the most powerful tools that Gobind’s group developed for investigating the BR proton pump mechanism was the ability to genetically engineer mutants of BR (site-directed mutagenesis) and express these mutants in a functional form. This required the development of many novel approaches that served as an important blueprint for future investigations of other integral membranes proteins. Many of these methods were published in a seminal series of 5 papers in JBC in 1987 (Braiman et al. 1987a, b; Dunn et al. 1987; Hackett et al. 1987; Karnik et al. 1987; Nassal et al. 1987). Part of this groundbreaking work involved Mark Braiman (Braiman et al. 1987a, b), now at the University of Syracuse, who was a joint post-doc in my lab at BU and Gobind’s at MIT and who played a major role in our collaboration aimed at applying these methods along with advanced vibrational techniques to investigate BR.

**Fig. 1:**
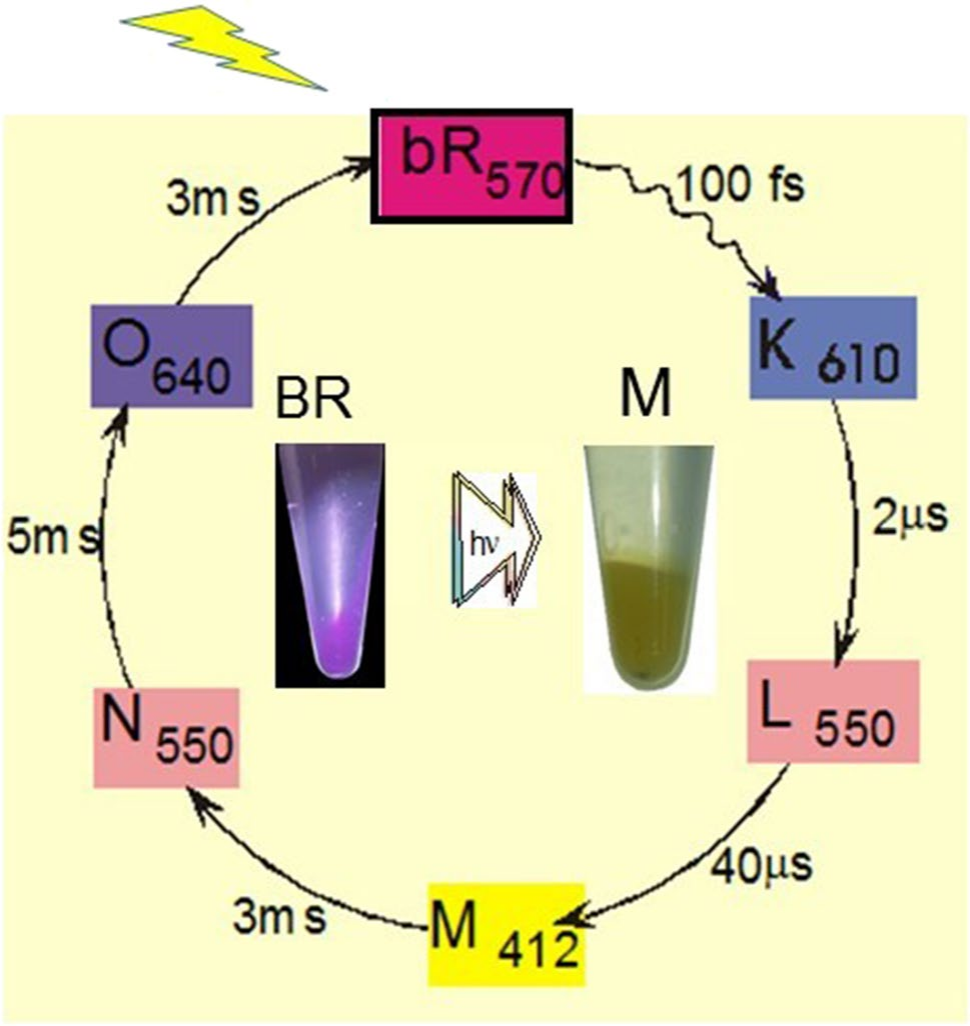
The BR Photocycle: After absorption of a photon (yellow arrow) by light-adapt BR_570_, the photocycle consists of a series of intermediate states with different characteristic life-times that ultimately results in the returns back to the original light-adapted BR_570_ state. Subscripts designate the wavelength of maximum visible absorption of each intermediate in the photocycle. Centeral image shows the color of the BR and M states. A key goal of our collaboration with the H.G. Khorana group was to elucidate conformational changes occurring in BR during each step in this photocycle (from reference Rothschild (2016))

Our major goal was to characterize the conformational changes in BR and its mutants at different steps during the BR photocycle (Fig. 1) using FTIR difference spectroscopy. The technique was first introduced by my laboratory to study BR in 1981 and later vertebrate rhodopsin in 1983 (Rothschild et al. 1981; Rothschild and Marrero 1982; Rothschild et al. 1983). This approach was also quickly adopted by several other biophysics laboratories (Bagley et al. 1982; Siebert and Mantele 1983; Gerwert 1992 Brown et al. 1995). The bands in FTIR difference spectra of BR reflect changes in the vibrational spectrum that are triggered by light absorption and occur during the different steps in the BR photocycle (Fig. 1). These bands appear in the difference spectrum of BR due to small changes in the structure of the protein, retinylidene chromophore and even local changes of specific amino acids including protonation changes and alterations in hydrogen bonding (Rothschild 1986, 1992; Braiman and Rothschild 1988).

**Fig. 2:**
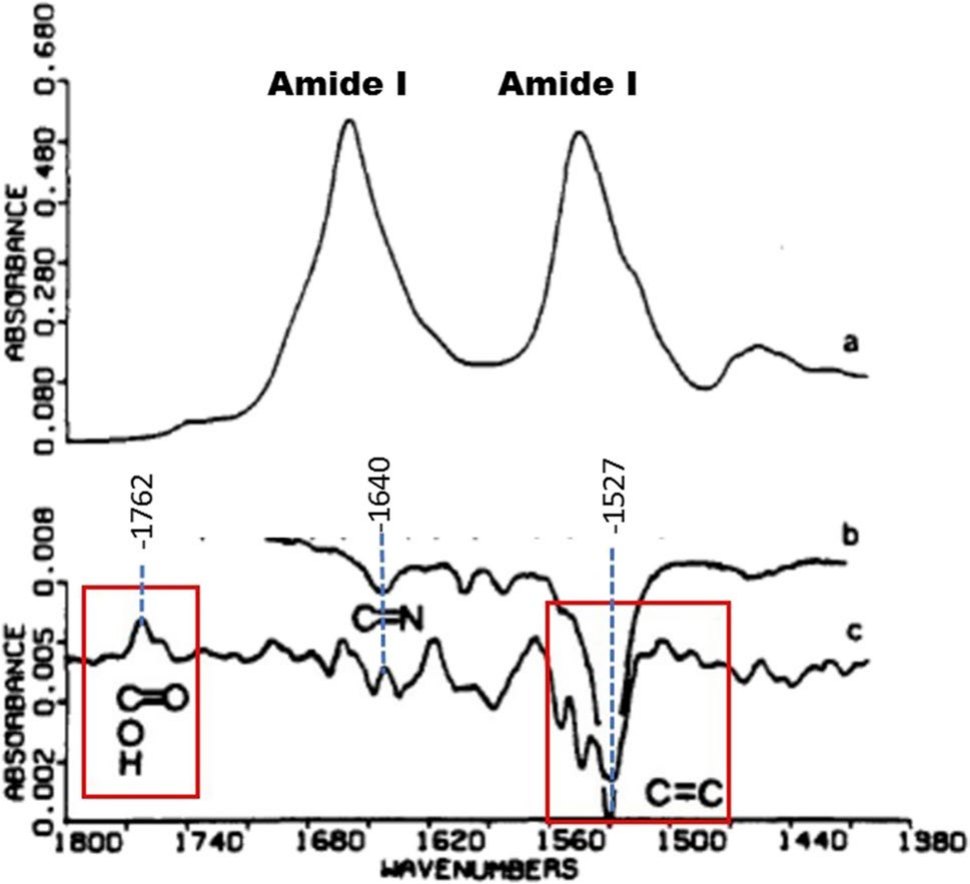
The first FTIR difference spectrum recorded of an integral membrane protein. While the FTIR absorption spectrum of purple membrane reveals little about the mechanism of BR proton transport (*top trace*), the difference spectrum is rich in information about protein and chromophore groups that undergo a structural change during the BR to M phototransition (*bottom trace*). The band at 1762 cm.^−1^ was associated with a protonation of an Asp or Glu residue (red box), whereas other bands were associated with chromophore groups (red box) based on comparison to resonance Raman spectra (*middle trace*) (adapted from reference Rothschild et al. (1981))

In the first reported BR FTIR difference spectrum, which measured the BR_570_ to M_412_ transition in dehydrated purple membrane films which slows the BR photocycle (Rothschild et al. 1981) (see Fig. 1), characteristic bands were identified arising from the retinylidene chromophore and protein (Fig. 2). Of particular interest were bands in the carboxylic C = O stretch region from 1700 to 1800 cm^−1^. The positive band at 1762 cm^−1^ was predicted to reflect the protonation of an Asp or Glu residue, possibly associated with the deprotonation of the retinylidene Schiff base upon formation of the M_412_ intermediate. In addition, negative bands were found at 1640 cm^−1^ (C = N stretch of protonated Schiff base) and at 1527 cm^−1^ (C = C ethylenic stretch of 13-cis retinal). While this newfound ability to detect small conformational changes in proteins using FTIR difference spectroscopy was very exciting, the challenge remained of how to relate these bands to specific amino acid residues in the protein which underwent structural changes at different steps in the photocycle.

Some progress in this direction was made between 1981 and 1988 by us and other groups using isotope labeling methods which shifts FTIR difference bands due to isotope effects ((Rothschild 1988) and references therein). One example is the approach from the Seibert group and colleagues who isotope labeled aspartic acid residues in BR (Engelhard et al. 1985). Much of this work by our group was facilitated by a close collaboration with Judy Herzfeld who developed methods for stringent isotope labeling of BR in *H. Salinarum* including, for example, tyrosine, tryptophan, and proline (Roepe et al. 1987a, 1987b, Roepe et al. 1987a, 1987b, Roepe et al. 1988a, b, Roepe et al. 1988a, 1988b, Rothschild et al. 1989a, b). Additional progress was made by the development of low-temperature, time-resolved, ATR and polarized FTIR difference methods which allowed us to resolve changes during different steps in the BR photocycle and under different conditions (Braiman and Rothschild 1988). However, the identity of specific amino acid residues in the BR sequence which gave rise to these bands remained largely unknown. This limitation posed a major challenge and a key to understanding the BR proton pump mechanism. The ability developed by Gobind’s group to perform site-directed mutagenesis of BR not only opened the door for detailed structure–function studies but provided a powerful method to assign bands to specific amino acids in the FTIR difference spectrum.

The first application of this new approach involved low-temperature FTIR difference measurements of a complete series of 11 Tyr to Phe mutations, expressed in *E. coli* and subsequently refolded by adding retinal and *H. Salinarum* polar lipids into reconstituted membranes (Braiman et al. 1988a, b). Out of the 11 tyrosines, the Tyr185 to Phe substitution was clearly identified as causing the disappearance of a set of previously identified bands in the BR to K and BR to M difference (Roepe et al. 1988a, b).

**Fig. 3:**
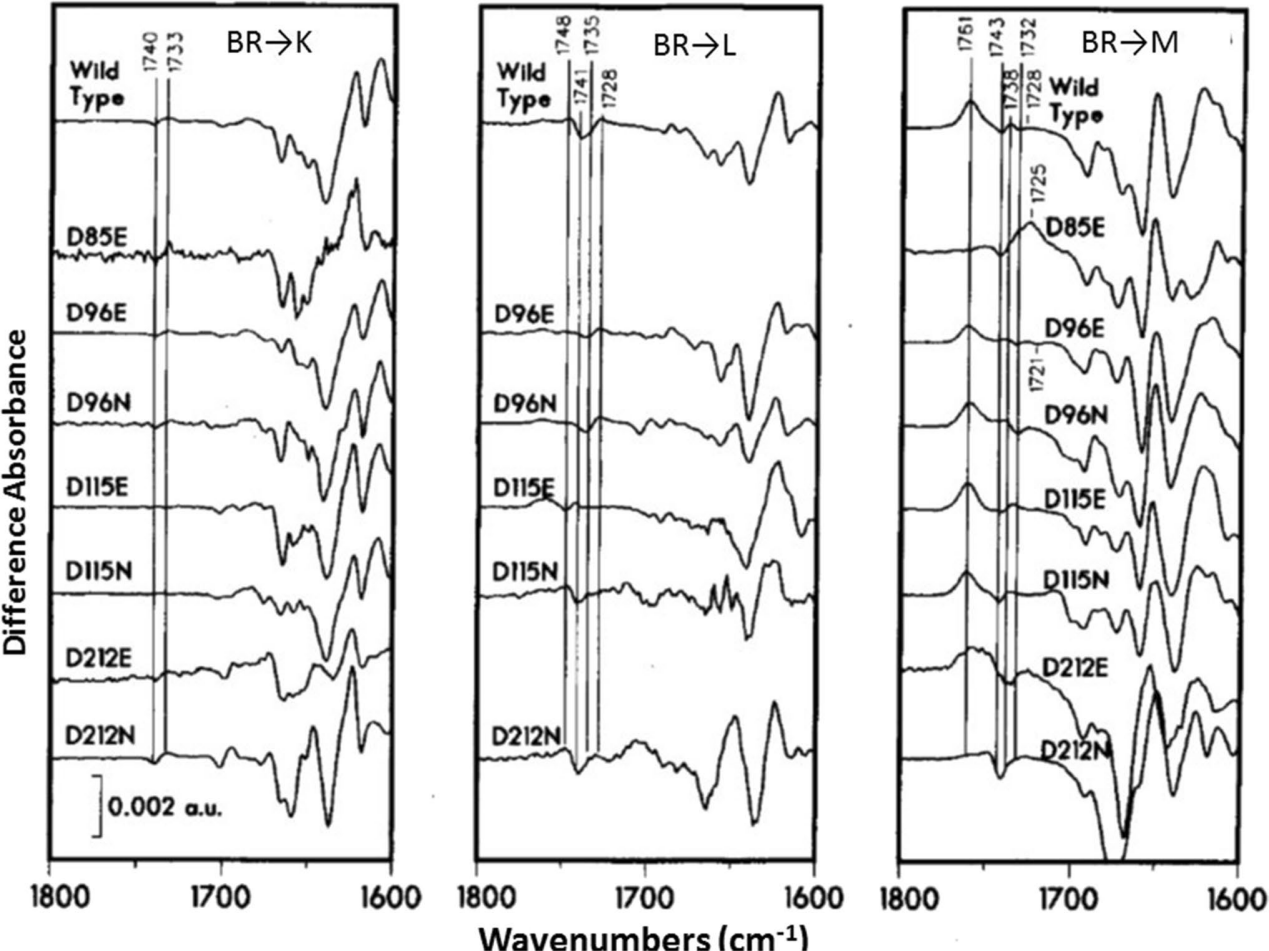
Comparison of FTIR difference spectra for WT and Asp → Asn, Glu substations in the BR amino acid sequence for the BR → K, L and M transitions recorded at low-temperature. Additional low-temperature studies on the BR → N (Bousche, Braiman et al. 1991) and BR → O transitions (Bousche et al. 1992), as well as time-resolved FTIR difference measurements (Braiman, Ahl et al. 1987b, a, Braiman, Bousche et al. 1991a, b, Bousche et al. 1992), led to an early model of the BR proton pump mechanism (adapted from Braiman et al. (1988a, b))

Using a similar approach published in 1988, bands in the carboxylic acid C = O stretch region (see Fig. 2) could be assigned to 4 key aspartic acid residues (Asp85, 96, 115, and 212) in the low-temperature FTIR difference spectrum for the BR to K, L and M photoreactions (Braiman et al. 1988a, b) (Fig. 3). Most prominently, the positive band at 1762 cm^−1^ (Fig. 2) was assigned to Asp85 and associated with the uptake of a proton from the Schiff base, while the negative/positive pair of peaks at 1742/1748 cm^−1^ was assigned to a change in hydrogen bonding of Asp96 during the L and M steps in the photocycle. A tentative model of the proton pump was proposed on this basis and additional structure–function studies on mutants performed in Gobind’s lab. One key feature of this model postulated that proton ejection involved a small movement of Arg82 in the direction of the cytoplasmic side of the membrane due to disruption of its interaction with Asp85 which is neutralized upon protonation and formation of the M_412_ intermediate. In fact such a movement was detected about 15 years later by x-ray crystallography (Luecke et al. 1999a, b; Luecke et al. 1999a, b).

In subsequent work involving Gobind’s group we showed that in the next step in the photocycle, the formation of the N intermediate (Fig. 1), Asp96 underwent complete deprotonation, coincident with reprotonation of the Schiff base (Bousche, Braiman et al. 1991). In addition, using a combination of time-resolved FTIR and site-directed mutagenesis we found that Asp96 reprotonates during O formation, while Asp85 deprotonates during O decay (Bousche, Spudich et al. 1991a, b; Bousche et al. 1992). A model which synthesized many of these features and incorporated high-resolution electron diffraction results (Henderson et al. 1990) was published by us in 1994 that also incorporated data from site-directed isotope labeling (Sonar et al. 1994).

There were many other examples where our collaboration with Gobind’s group provided early insight into the mechanism of BR. These include work with Sriram Subramaniam on the role of Leu93 in retinal isomerization and photocycle kinetics (Subramaniam et al. 1991a, b; Subramaniam et al. 1991a, b); and the elucidation of the role of other amino acids in the BR photocycle including Trp86 (Rothschild et al. 1989a, b); Thr46 and Thr89 (Rothschild et al. 1992, Rothschild et al. 1993); along with the detection of specific water molecules (Fischer et al. 1994). In general, this initial work helped establish some of the key features of the BR proton transport mechanism which was expanded on by other groups using a similar approach. One example is elucidation of the proton release mechanism at the cytoplasmic surface which was shown to involve a complex formed by Asp194 and Asp204 (Brown et al. 1995). Another is the detection of individual water molecules that are involved in the BR proton pump mechanism and their interaction with specific residues (Fischer et al. 1994; Maeda et al. 1997; Garczarek and Gerwert 2006, Lorenz-Fonfria et al. 2008). Overall, there are hundreds of additional examples of the combined application of FTIR difference spectroscopy and site-directed mutagenesis to the study of BR, other Type I and II rhodopsins and membrane proteins in general.

Gobind’s life-long approach to rigorous science, willingness to tackle the “hard stuff” and ability to plunge himself into the smallest details of the experiments, often working directly at the lab bench was an important inspiration for all of us during all phases of our collaboration. I fondly remember the weekly group meetings and lunches at MIT where we had discussed in detail each phase of the research. Gobind would often walk from MIT across the Harvard-MIT bridge to make a surprise visit to our laboratory at BU in the Science and Engineering Center. This work involved many members of Gobind’s group that I got to know well including Hagan Bayley, Betty Chao, Mark Krebs, Neil Hackett, Kuo-Sen Huang, Erwin London, Tom Marti, Tatsushi Mogi, Larry Stern, and Sriram Subramaniam. Key members of my group who were involved in this collaboration and made important contributions included Pat Ahl, Pramod Argade, Steve Berkowitz, Mark Braiman, Olaf Bousche, Matt Coleman, Mireia Duñach, Tom Earnest, Wolfgang Fischer, Dan Grey, John Gillespie, Yi-Wu He, Xiaomei Liu, Cheryl Ludlam, Gary Ludlam, Hector Marrero, Jerzy Olejnik, Nilam Patel, Parshuram Rath, Paul Roepe, and Sanjay Sonar. We also worked closely during this period with Tom RajBhandary and Chan-Ping Lee in his group on developing advanced methods of protein engineering including site-directed isotope labeling using suppressor tRNA aminoacylated with an isotopically labeled amino acid (Sonar et al. 1994). My future collaborations with John Spudich’s group at the University of Texas McGovern Medical School starting in 1988 were also catalyzed by my collaboration with Gobind when John and Elena Spudich who did a sabbatical in his lab. The collaboration with John based in part on techniques established in Gobind’s lab led us to explore the mechanism of many other microbial rhodopsins such as halorhodopsin (Rothschild et al. 1988), sensory rhodopsins (Bergo et al. 2000), neurospora rhodopsin (Bergo et al. 2002); proteorhodopsin (Rothschild et al. 1988; Bergo et al. 2004) and more recently channelrhodopsins (Ogren et al. 2014; Yi et al. 2017; Li et al. 2021) using a combination of site-directed mutagenesis, FTIR difference spectroscopy and other biophysical techniques.

In general, the development of many of the innovative methods to study BR including site-directed mutagenesis that occurred during this period was a key enabler for us and other groups to apply FTIR difference spectroscopy to BR and other membrane proteins. This influence continues to have a major impact in the field of biophysics. On a personal level, my interactions with Gobind are fondly remembered and inspirational.

## Data Availability

Not applicable.
